# LncRNA PVT1 promotes exosome secretion through YKT6, RAB7, and VAMP3 in pancreatic cancer

**DOI:** 10.18632/aging.103268

**Published:** 2020-06-04

**Authors:** Chengming Sun, Peng Wang, Wei Dong, Haishi Liu, Jianmin Sun, Liang Zhao

**Affiliations:** 1 Department of Hepatopancreatobiliary Surgery, Harbin Medical University Cancer Hospital, Harbin 150081, Heilongjiang, China

**Keywords:** pancreatic cancer, exosome secretion, PVT1, YKT6, RAB7

## Abstract

Pancreatic cancer (PC) is one of the deadliest cancers worldwide. Cancer cells secrete excessive numbers of exosomes that play essential roles in tumorigenesis. Long non-coding RNAs (lncRNAs) are essential non-coding RNAs for cancer progression. However, the role of lncRNA plasmacytoma variant translocation 1 (PVT1) in exosome secretion of PC remains to be comprehensively investigated. Thus, nanoparticle tracking analysis and transmission electron microscopy were performed to determine exosome secretion. Confocal microscopy, western blots, real-time PCR, immunofluorescence, pull-down and RNA immunoprecipitation assays, and rescue experiments were applied to investigate the mechanism underlying the role of PVT1 in exosome secretion. The results showed that PVT1 was upregulated in PC cells, along with increased levels of YKT6 v-SNARE homolog (YKT6), ras-related protein Rab-7 (RAB7), and vesicle-associated membrane protein 3 (VAMP3). Also, PVT1 promoted the transportation of multivesicular bodies (MVBs) towards the plasma membrane. In addition, PVT1 promoted the docking of MVBs by altering RAB7 expression and localization. Moreover, PVT1 promoted the fusion of MVBs with the plasma membrane through regulating YKT6 and VAMP3 colocalization and the palmitoylation of YKT6. Taken together, the results suggest that PVT1 promoted exosome secretion of PC cells and thus, can expand the understanding of PVT1 in tumor biology.

## INTRODUCTION

Pancreatic cancer (PC) is one of the most devastating and fatal malignancies with poor prognosis and high mortality worldwide, manifesting the close parallel correlation between incidence and mortality [1]. Currently, less than 10% of patients with PC are diagnosed at an early phase. Therefore, most of the patients lack the opportunity to receive surgical treatment due to being diagnosed at a later phase [2]. The high mortality is primarily attributed to several factors, for example, family history, genetics, cigarette consumption, and chronic pancreatitis [1]. The key reason for high mortality is the advanced stage at which most patients are diagnosed [3]. Another challenge for PC treatment is that patients with PC respond poorly to either radiotherapy or chemotherapy [4]. The only curative treatment of PC is surgical resection; however, only 15% of tumors are suitable for resection due to late diagnosis [5]. As such, the exploration of the mechanism underlying the pathology and progression of PC is urgently needed.

Long non-coding RNAs (lncRNAs) are a class of non-coding RNAs containing more than 200 nucleotides in length and cannot encode functional proteins in the nucleus or cytoplasm [6]. Even though the mechanism and function of lncRNAs have not been comprehensively investigated, growing evidence indicates that lncRNAs are essential for the regulation of gene expression at both transcriptional and post-transcriptional levels [6, 7], thus playing crucial roles in various biological and pathological processes, including cancers [8–10]. Numerous reports suggest that lncRNAs act as crucial regulators for tumorigenesis of PC and as promising biomarkers for PC [11]. Among these studied lncRNAs, lncRNA plasmacytoma variant translocation 1 (PVT1) is one of the most well-documented cancer-associated regulators in several cancer types [12]. For example, PVT1 is found to be upregulated in PC and can serve as an independent prognostic factor for poor OS in PC patients [13, 14]. Also, the overexpression of PVT1 in the antisense orientation reconstituted can sensitize human ASPC-1 PC cell to Gemcitabine [15]. Furthermore, PVT1 has been reported to promote proliferation and migration of PC cells via acting as an endogenous sponge to compete with microRNA-448 for binding to SERPINE1 MRNA Binding Protein 1 [16].

The tumor microenvironment is critically essential to the development and progression of cancer [17]. As critical components in the tumor microenvironment, exosomes, double-layered microvesicles with a diameter of 50–100 nm, play an essential role in tumor development, such as angiogenesis, metastasis, migration, and chemoresistance [18–20]. Exosomal contents can also be applied as an early diagnostic biomarker and to monitor tumor progression [21]. In addition, a growing number of studies suggest that exosomes derived from cancer cells can carry bioactive cargos, including enzymes, miRNAs, and lncRNAs, to neighboring cells, ultimately regulating tumorigenesis [22, 23]. Collectively, exosomes have emerged as novel promising targets for diagnostic and therapeutic applications in cancers. Because of this, there is a large amount of attention to investigate the mechanism underlying the secretion of exosomes from tumor cells [24–26]. As a complex multi-step process, the secretion of exosomes is associated with multivesicular bodies (MVBs) transportation, docking, and fusion with plasma membranes, which are regulated by several related molecule families, such as RAB and SNARE [27, 28]. However, the function of lncRNAs in the secretion of cancer exosomes still needs to be fully elucidated. Therefore, based on the previous studies mentioned above, we aimed to investigate whether PVT1 contributes to exosome secretion of PC and to elucidate its related signaling pathways.

## RESULTS

### Upregulation of PVT1 is associated with exosome secretion

**Figure 1:**
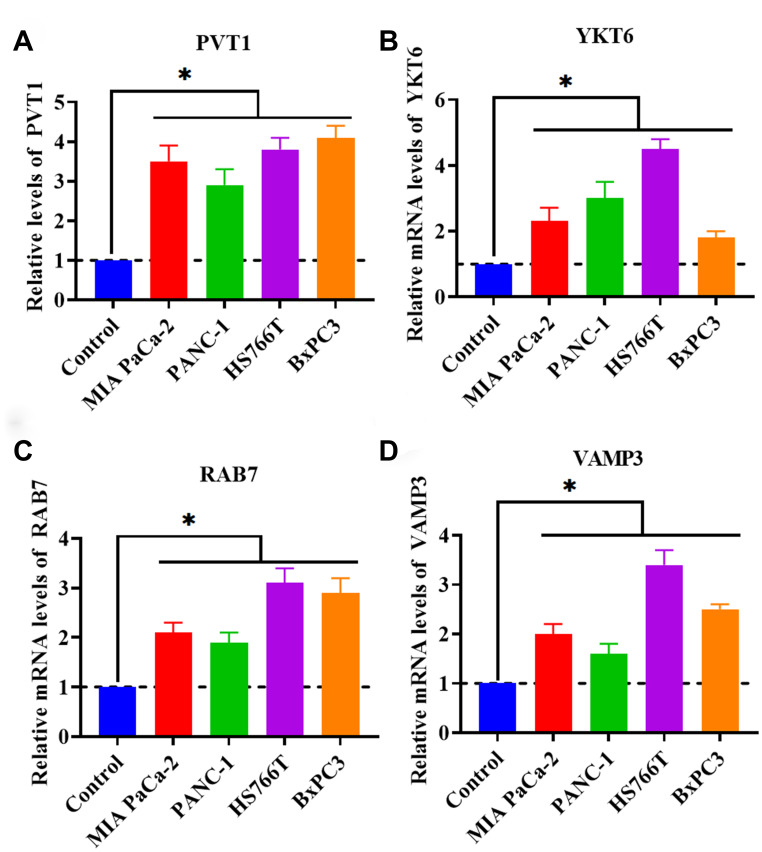
**PVT1 and exosome secretion-associated factors are increased in PC cell lines.** (**A**) The expression of PVT1 in PC cell lines. (**B**) The mRNA expression of YKT6 in PC cell lines. (**C**) The expression of RAB7 in PC cell lines. (**D**) The expression of VAMP3 in PC cell lines. *P < 0.05, data are expressed as the mean ± SD.

As mentioned in the introduction section, PVT1 has been demonstrated to act as an important regulator in PC. However, the role of PVT1 in exosome secretion has not been reported in PC. Thus, in this study, we proposed to investigate the correlation between exosome secretion and PVT1. We first determined the expression level of PVT1 in PC cell lines (MIA PaCa-2, PANC-1, HS766T, and BxPC3). The results obtained from qRT-PCR revealed that PVT1 was increased in all four tested PC cell lines (Figure 1A), which was consistent with previous findings [14, 16, 29]. Meanwhile, we detected the mRNA levels of several key regulators associated with the process of exosome secretion, including RAB2B, RAB5, RAB7, RAB9A, RAB11, RAB27A, RAB27B, RAB35, VAMP3, VAMP7, SNAP23, and YKT6. In addition, the results showed that the expressions of YKT6, RAB7, and VAMP3 were upregulated in PC cells (Figure 1B–1D). Collectively, these findings suggested that the secretion of exosomes may be increased in PC cells compared with healthy control cells and that the upregulation of PVT1 may be associated with increased exosome secretion.

### Upregulation of PVT1 stimulates exosome secretion of PC HS766T cells

**Figure 2:**
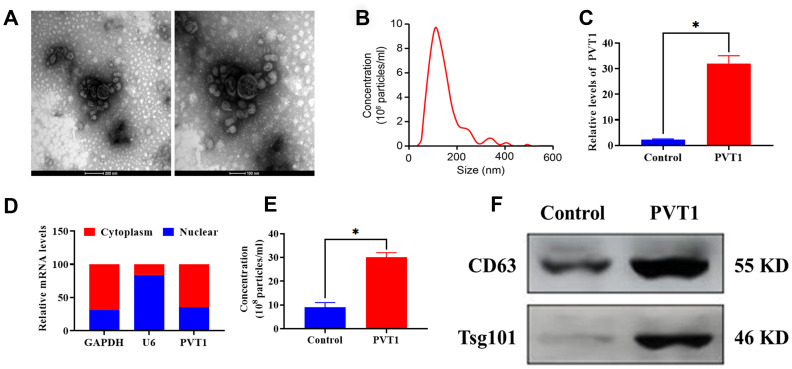
**PVT1 stimulates exosome secretion in HS766T cells.** (**A**) Representative images of exosomes derived from HS766T cells, as detected by TEM. Scale bar: 200 nm (left) and 100 nm (right). (**B**) The size distribution of exosomes, as determined by NTA. (**C**) The transfection efficiency of PVT1 overexpression in HS766T cells. (**D**) The expression of PVT1 in the nucleus and cytoplasm of HS766T cells. (**E**) The concentration of exosome derived from PVT1-overexpressing HS766T cells. (**F**) The protein expression of exosome markers in PVT1-overexpressing HS766T cells. *P < 0.05, data are expressed as the mean ± SD.

Exosomes derived from PC HS766T cells were isolated from cell culture medium via the ultracentrifugation assay. As shown in Figure 2A, 2B, the morphology of exosomes exhibited a round-shape and double-membrane with a diameter of 50 - 120 nm. Next, HS766T cells were transfected with pcDNA3.1-PVT1 vectors to overexpress PVT1. The efficiency of transfection was determined by qRT-PCR, and the results showed that the expression of PVT1 displayed a 15-fold increase compared with the control group (Figure 2C). Also, PVT1 was found to be primarily expressed in the cytoplasm of HS766T cells, relative to the nucleus (Figure 2D). According to the NTA, the overexpression of PVT1 was associated with increased exosome secretion (Figure 2E), which was also verified by elevated protein expression of exosome markers, CD63 and Tsg101, in exosomes derived from HS766T cells, compared with those derived from control cells (Figure 2F). Thus, these results indicated that the overexpression of PVT1 might exert a positive role in exosome secretion of PC cells.

### Upregulation of PVT1 promotes the movement of MVBs towards the plasma membrane

**Figure 3:**
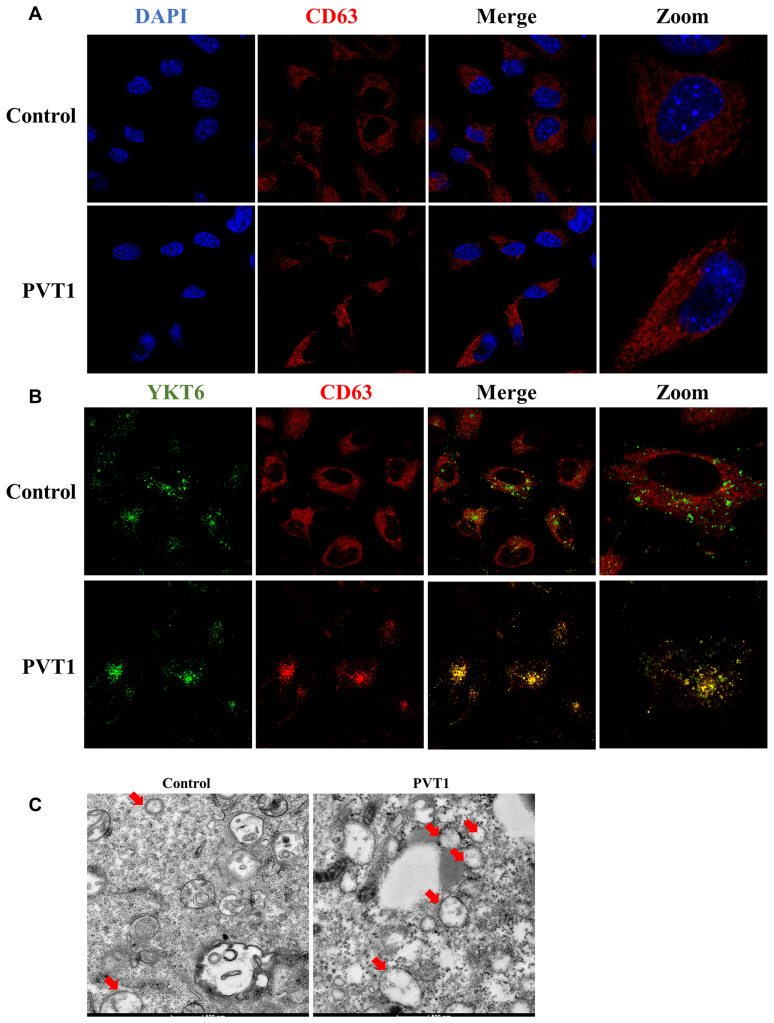
**PVT1 promotes the movement of MVBs towards the plasma membrane.** (**A**) Analysis of CD63 (red) in PVT1-overexpressing HS766T cells, as determined by confocal microscope. Nuclei were labeled with DAPI (blue). (**B**) Analysis of YKT6 (green) and CD63 (red) in PVT1-overexpressing HS766T cells, as determined by confocal microscope. (**C**) Exosomes in PVT1-overexpressing HS766T cells, as determined by electron microscope.

It has been demonstrated that exosome secretion is involved in several important steps, including MVBs transportation, docking, and fusion with the plasma membrane [27, 28]. Thus, to determine the transportation of MVBs that contain exosomes, CD63 was used as a marker to label MVBs. As shown in Figure 3A, the overexpression of PVT1 led to MVBs to move far away from the nucleus. Moreover, YKT6 is an essential member of the SNARE complex that plays an essential role in MVBs docking and fusion with the plasma membrane [27, 28]. In this study, confocal colocalization analysis suggested that the increase of PVT1 in HS766T cells resulted in more colocalization of CD63 and YKT6, suggesting MVBs moved towards the plasma membrane (Figure 3B). Furthermore, more MVBs were observed in PVT1-overexpressing HS766T cells using the electron microscope, compared with the control cells (Figure 3C). Together, these findings suggested that PVT1 may play an essential role in the transportation of MVBs towards the plasma membrane.

### PVT1 affects the expression and localization of RAB7 in HS766T cells

**Figure 4:**
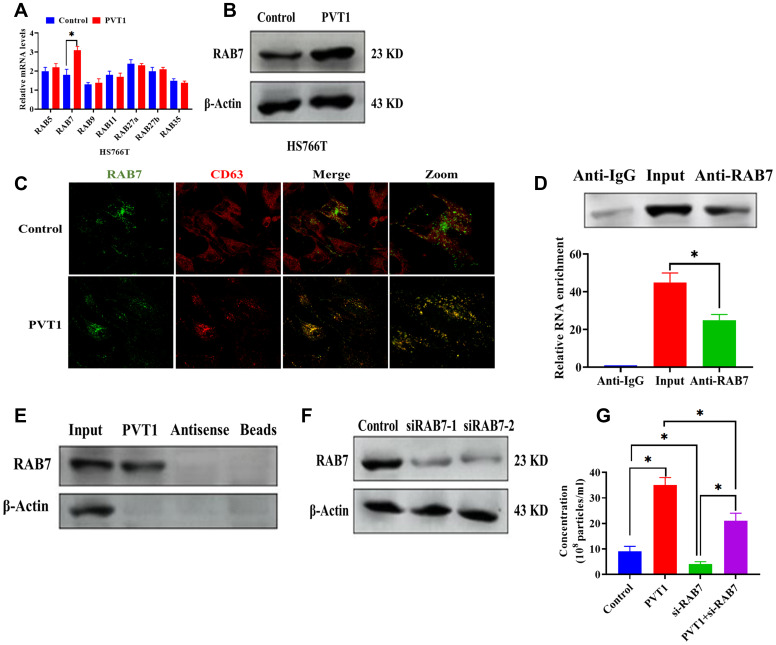
**PVT1 affects the expression and localization of RAB7 in HS766T cells.** (**A**) The mRNA expression of Rab GTPases genes in PVT1-overexpressing HS766T cells. (**B**) The protein expression of RAB7 in PVT1-overexpressing HS766T cells. (**C**) Analysis of RAB7 (green) and CD63 (red) in PVT1-overexpressing HS766T cells, as determined by confocal microscope. (**D**) The interaction between PVT1 and RAB7, as determined by RIP assay and qRT-PCR. (**E**) The correlation between PVT1 and RAB7, as determined by pull-down assay. (**F**) The knockdown efficiency of si-RAB7 in HS766T cells. (**G**) The concentration of exosome derived from HS766T cells with overexpression of PVT1 and knockdown of RAB7. *P < 0.05, data are expressed as the mean ± SD.

To further determine the role of PVT1 in the transportation of MVBs, we first detected the mRNA expressions of several RAB GTPases in PVT1-overexpressing HS766T cells (Figure 4A). The results demonstrated that both mRNA and protein expressions of RAB7 were upregulated in HS766T cells transfected with pcDNA3.1-PVT1 vectors while the overexpression of PVT1 did not affect the expressions of other RAB GTPases (Figure 4A and 4B). By confocal colocalization analysis, we found that the overexpression of PVT1 promoted the colocalization of RAB7 with CD63 (Figure 4C), indicating that PVT1 is associated with the distribution of RAB7. In addition, results obtained from the RIP assay showed that PVT1 was highly enriched by the RAB7 antibody (Figure 4D). Also, the pull-down assay revealed a physical interaction between PVT1 and RAB7 (Figure 4E). These results together suggest a direct interaction between PVT1 and RAB7. To further study the role of RAB7 in exosome secretion, we applied si-RAB7 to knockdown RAB7, and the efficiency of si-RAB7 was determined by western blots (Figure 4F). According to the NTA, we found that the knockdown of RAB7 significantly inhibited exosome secretion while the overexpression of PVT1 abolished the effect of si-RAB7 in exosome secretion (Figure 4G).

### PVT1 regulates the translocation of YKT6 and VAMP3

**Figure 5:**
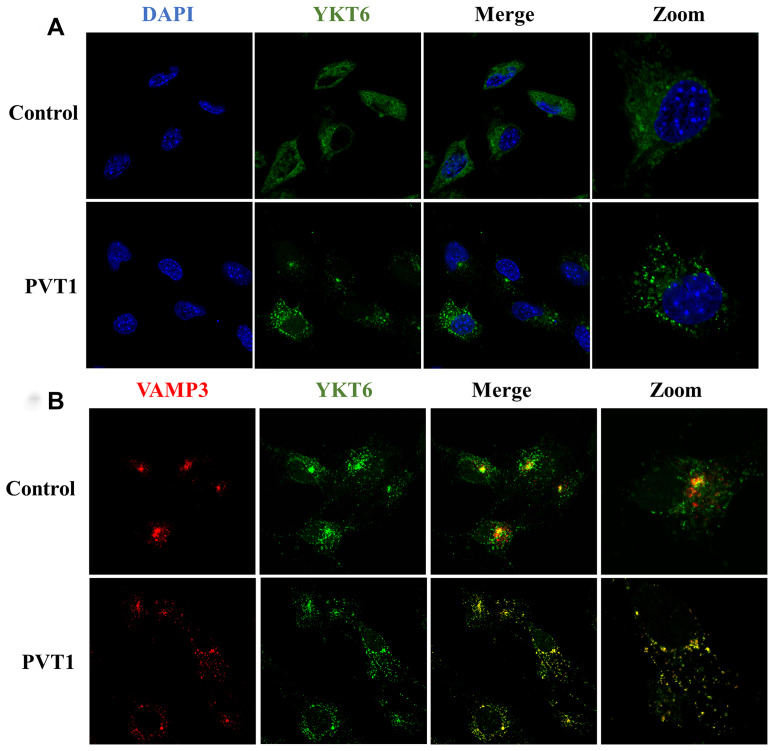
**PVT1 regulates the translocation of YKT6 and VAMP3.** (**A**) Analysis of YKT6 (green) in PVT1-overexpressing HS766T cells, as determined by confocal microscope. Nuclei were labeled with DAPI (blue). (**B**) Analysis of YKT6 (green) and VAMP3 (red) in PVT1-overexpressing HS766T cells, as determined by confocal microscope.

During the process of fusion of MVBs with the plasma membrane, soluble N-ethylmaleimide-sensitive factor attachment protein receptors (SNAREs) family proteins are a class of essential regulators for exosome release [30, 31]. As an essential member of the SNARE complex, YKT6 was increased in PVT1-overexpressing HS766T cells and moved away from the nucleus (Figure 5A). Meanwhile, the overexpression of PVT1 increased the colocalization of YKT6 and VAMP3 (Figure 5B), which is reported as a key v-SNARE molecule [32]. Collectively, the results indicated that PVT1 might promote the formation of the SNARE complex and then facilitate the fusion of MVBs with the plasma membrane.

### PVT1 stimulates exosome secretion via palmitoylation of YKT6

**Figure 6:**
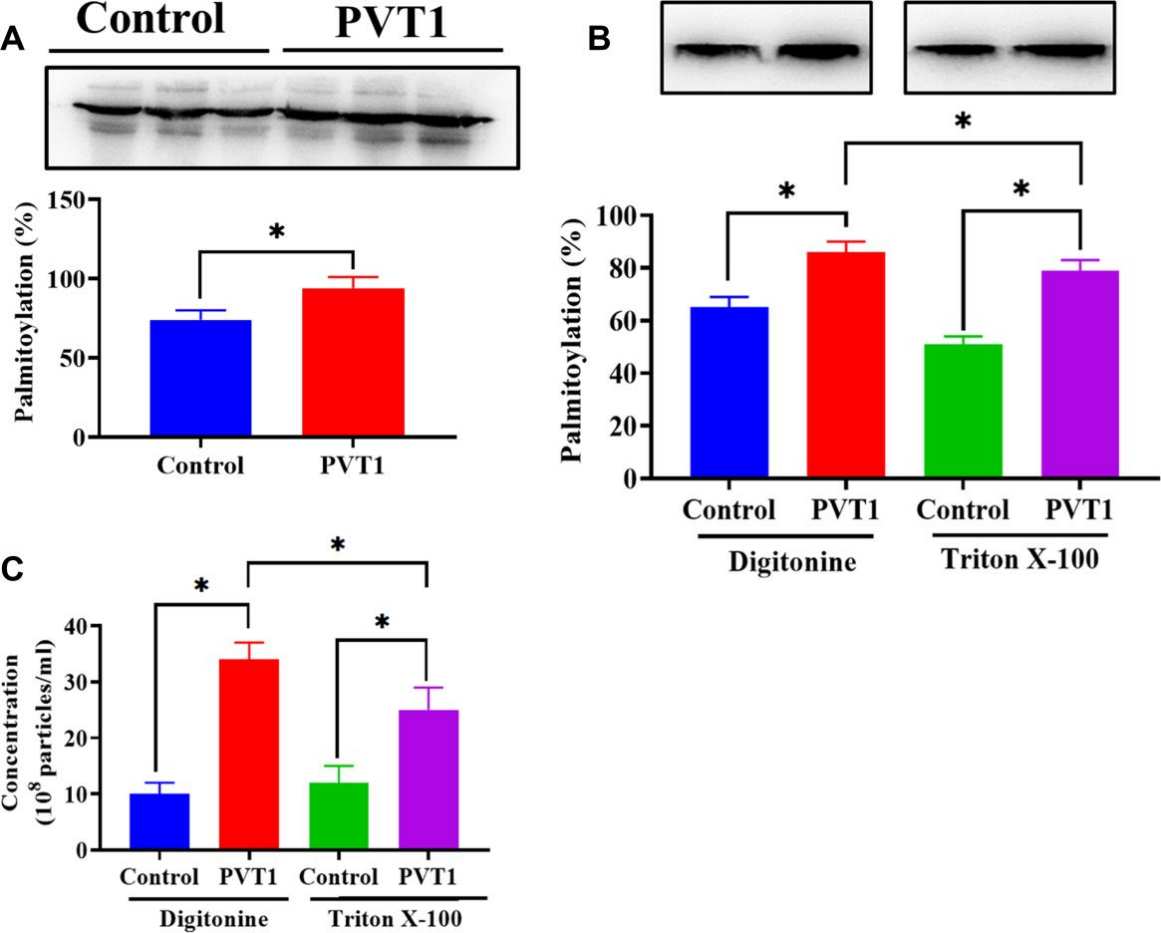
**PVT1 stimulates exosome secretion via palmitoylation of YKT6.** (**A**) The level of palmitoylation of YKT6 in PVT1-overexpressing HS766T cells. (**B**) The level of palmitoylation of YKT6 in PVT1-overexpressing HS766T cells treated with Digitonine (control) or Triton X-100 (palmitoylation inhibitor). (**C**) The concentration of exosomes derived from PVT1-overexpressing HS766T cells treated with Digitonine (control) or Triton X-100 (palmitoylation inhibitor). *P < 0.05, data are expressed as the mean ± SD.

It has been reported that the activity of YKT6 is associated with the level of palmitoylation [33, 34]. Thus, according to the palmitoylation assay, we found that the palmitoylation level of YKT6 was significantly increased in PVT1-overexpressing HS766T cells (Figure 6A). The addition of Triton X-100, a palmitoylation inhibitor [35], decreased palmitoylation of YKT6, compared with the control group (Figure 6B). Furthermore, decreased palmitoylation of YKT6 was associated with less exosome secretion in PVT1-overexpressing HS766T cells (Figure 6C). Thus, our results demonstrated that PVT1 might stimulate exosome secretion through the palmitoylation of YKT6.

## DISCUSSION

Pancreatic cancer has emerged as one of the leading causes of cancer-associated death all over the world [36]. In the past decade, the incidence of PC increased 3-fold, ranking it the seventh disease of cancer mortality in China and the fourth worldwide [37, 38]. Even though the biological knowledge of PC has been dramatically increased in the past decades, the leading causes of PC remain to be investigated, and better diagnosis and therapeutic strategies continue to be of urgent need. Given the importance of exosomes in tumorigenesis and cancer progression [18–20], the present study found that PVT1 could facilitate the secretion of exosomes from PC cells. Also, PVT1 exerts a positive role in the transportation of MVBs towards the plasma membrane and the docking process through RAB7. Furthermore, PVT1 promotes the colocalization of YKT6 and VAMP3, resulting in the fusion of MVBs with the plasma membrane.

Growing evidence reports that lncRNAs participate in a wide range of biological activities, and aberrant expression of lncRNAs is associated with many pathological processes, such as cancers [39]. To date, numerous studies have revealed that lncRNAs play important roles in various tumor cell activities, including metastasis, invasion, migration, proliferation, and drug resistance [39, 40]. In particular, the function of PVT1 has been reported in various cancer types and summarized in several review papers [12, 41, 42]. In PC, PVT1 can be applied as a potential biomarker for predicting the prognosis [14]. Also, PVT1 is essential for Gemcitabine sensitivity, proliferation, migration, and epithelial-mesenchymal transition in PC cells [16, 29, 43]. However, the role of PVT1 in exosome secretion of PC cells has not been fully studied. In this study, we found that the overexpression of PVT1 promoted exosome secretion in PC cells, along with increased transportation of MVBs, docking, and fusion with the plasma membrane. Therefore, our findings indicated that there is a positive correlation between the level of PVT1 and exosome secretion in PC cells.

Exosomes are crucial intercellular communication modes to transmit molecular information, including proteins, enzymes, and non-coding RNAs between cancer cells and between cancer cells and the tumor stroma [44]. Also, cancer cells can release a large number of exosomes compared to normal cells [45], suggesting that the active secretion of exosomes may be a key functional implication for cancer progression. Therefore, exosome secretion associated with this mechanism has drawn increasing scientific attention. In general, the process of exosome secretion is involved in several important intracellular trafficking steps, including the transportation of MVBs, docking, and fusion with the plasma membrane [28]. During this series of processes, the RAB GTPase family is essential for the transportation of MVBs and docking at the plasma membrane [46, 47], of which several RAB GTPases has been demonstrated to act as key regulators in the exosome-associated function as well as exosome release, such as RAB5, RAB7, RAB27A, RAB27B, and RAB35 [27, 48]. In the present study, we determined the mRNA expressions of several RAB GTPase factors and found RAB7 to be significantly upregulated in all four PC cell lines, implying a potential role of RAB in exosome secretion of PC. Furthermore, the forced expression of PVT1 not only increased the level of RAB7 but also promoted RAB7 to be located at the membrane of MVBs. Vanlandingham et al. reported that RAB7 plays a vital role in the endocytic organelle maintenance and the cargo shuttling from the late endosome/MVB to the lysosome [49]. Also, RAB7 is critical for syntenin/ALIX-carrying exosome secretion in MCF-7 breast cancer cells [50]. Thus, these results suggest that the involvement of RAB7 may be required for PVT1-associated MVBs transportation.

After transportation and docking, MVBs fusion with the plasma membrane is the last phase of exosome secretion [28], in which the SNARE complex is an essential mediator [31]. As a member of the SNARE family, YKT6 is highly conserved across species and broadly distributed in the membrane, cytosol, and perinuclear locations [51]. Also, YKT6 has been identified as a critical protein in cell membrane fusion and vesicular transportation [52]. In the current study, we observed that PVT1 promote the diffused location of YKT6 at the plasma membrane, and the overexpression of PVT1 resulted in the colocalization of YKT6 with VAMP3, which is a critical SNARE protein for MVBs fusion [53, 54]. Previous evidence revealed that YKT6 is a crucial protein for the secretion of WNT3A-carrying exosome in HEK293 cells [55]. In lung cancer, the suppression of YKT6 remarkably inhibited exosome secretion in the NSCLC cell line [56]. Collectively, our findings suggested that PVT1 is associated with the formation of the SNARE complex to facilitate the fusion of MVBs with the plasma membrane.

There are some limitations that should be addressed in the future. First, our results showed that the expressions of PVT1, YKT6, RAB7, and VAMP3 were higher in all four PC cell lines, indicating that PVT1 may exert a similar effect on exosome secretion in other PC cell lines, not only in HS766T cells. Thus, future studies are necessary to address this issue. Second, the function of PVT1 in exosome secretion should also be verified in the xenograft mouse model, which will significantly extend the understanding of the mechanism underlying exosome secretion. Lastly, the final fate of exosomes that are not secreted to the extracellular environment can fuse with the lysosomes or autophagosomes, leading to degradation of their content [57]. In this study, we did not explore the mechanism underlying the balance between exosome secretion and degradation. In cancers, both exosome secretion and autophagy processes are activated in the development and progression of tumors [58, 59]; thus the further investigation of this balance will provide a more in-depth look into the physiology of tumor cells.

In conclusion, the results suggested that PVT1 plays a positive role in exosome secretion of PC cells by promoting the transportation of MVBs towards the plasma membrane, docking, and fusion. Also, SNARE proteins, YKT6, VAMP3, and GTPase RAB7, are essential for PVT1-mediated exosome secretion. This study extends our understanding of the function of PVT1 in PC and provides novel insight into the role of lncRNAs in tumor biology.

## MATERIALS AND METHODS

### Cell culture

Human Pancreatic Duct Epithelial Cell Line (H6C7) was purchased from Kerafast, Inc. (Boston, MA, USA) and cultured in Keratinocyte Basal Medium (Sigma-Aldrich, Shanghai, China). Human PC cell lines MIA PaCa-2, PANC-1, HS766T, and BxPC3, were purchased from American Type Culture Collection (ATCC; Manassas, VA, USA). The PC cell lines were maintained in Dulbecco’s Modified Eagle Medium (DMEM; Sigma-Aldrich, Shanghai, China) supplemented with 10% fetal bovine serum (Life Technologies, Grand Island, NY, USA) at 37°C with 5% CO_2_. The morphology, growth curve, and mycoplasma detection of PC cell lines were determined one month prior to the experiment, according to the cell line verification test recommendation from ATCC.

### Transfection

The PVT1-loaded plasmids, small interfering RNAs for RAB7 (siRAB7), and corresponding negative controls were purchased from Applied Biological Materials Inc. (Richmond, BC, Canada). The full-length cDNA sequence of PVT1 was inserted into the pcDNA3.1 vector to create the PVT1 overexpression plasmid. The two siRAB7 sequences were as following: siRAB7-1: 5′-TACGTCCAAGGTCGGGCAGGAAGA-3′ and siRAB7-2: 5′- TACGTCCAAGGTCGGGCAGGAAGA-3′. Cell transfection was performed using the Lipofectamine™ 3000 Reagent (Invitrogen, Carlsbad, CA, USA) according to the manufacturer’s instructions.

### Exosome isolation

Exosomes were isolated from the culture supernatant of HS766T cells using ultracentrifugation as previously described [60]. Exosomes were collected from the pellet and resuspended in PBS for subsequent experiments.

### Transmission electron microscopy (TEM)

Exosomes were placed on Formvar carbon-coated electron microscopy grids (Electron Microscopy Sciences, Hatfield, PA, USA) and incubated at room temperature for 5 minutes. Then, exosomes were fixed using 2% paraformaldehyde and washed with water twice. Next, the grids were stained with 10% uranyl acetate for 10 minutes. The morphology of exosomes was imaged using the JEOL 100XCII electron microscope (Peabody, MA, USA).

### Nanoparticle tracking analysis (NTA)

The size and number of exosomes were determined by the NanoSight NS300 system (Salisbury, UK) according to the manufacturer’s instructions. The exosome sample was diluted 150 - 3000 times using Dulbecco’s PBS to obtain a concentration of 1-20 × 10^8^ particles per milliliter.

### Quantitative real-time PCR (qRT- PCR)

Total RNAs were isolated from cells or exosomes using the TRIzol reagent (Invitrogen, Carlsbad, CA, USA) according to the manufacturer’s instructions. Reverse transcription was performed using the High-Capacity cDNA Reverse Transcription Kit (Thermo Fisher Scientific, Waltham, MA, USA). The PCR reaction was performed on the Bio-Rad Icycler Pcr Thermal Cycler (Hercules, CA, USA) using SYBR™ Green PCR Master Mix (Thermo Fisher Scientific, Waltham, MA, USA). Table 1 lists the primers used.

**Table 1:** Primer information.

**Gene name**	**Primer sequence**
**RAB2B (F)**	5′- GGTCCGGGAAGTCCATACTC -3′
**RAB2B ®**	5′- GGCTGGAACCGCTTATCTGT -3′
**RAB5 (F)**	5’- AGACCCAACGGGCCAAATAC -3’
**RAB5 ®**	5’- GCCCCAATGGTACTCTCTTGAA -3’
**RAB7 (F)**	5′- CTCATTATCGTCGGAGCCATTG -3′
**RAB7 ®**	5′- AGTGTGGTCTGGTATTCCTCATA -3′
**RAB9A (F)**	5’- GGCAACCTTGCGACTATAACCA-3’
**RAB9A ®**	5’- GTTTCCTCTCCCTGAGACCCTA-3’
**RAB11 (F)**	5′- GCTCGGCCTCGACAAGTTC -3′
**RAB11 ®**	5′- ACTTATACCACTGCGTCTTCCT -3′
**RAB27A (F)**	5’- GGAGAGGTTTCGTAGCTTAACG -3’
**RAB27A ®**	5’- CCACACAGCACTATATCTGGGT -3’
**RAB27B (F)**	5’- AGAAGCTCTGTTGACTGGTGA -3’
**RAB27B ®**	5’- GTTGGATCCTATTAATAGGGGGCCCATGCAAGAT-3’
**RAB35 (F)**	5’- TTAAGCTTCGATGGCCCGGGACTACGACC -3’
**RAB35 ®**	5’- TTGGATCCTTAGCAGCAGCGTTTCTTTCGTTTACTG -3’
**VAMP3 (F)**	5′- ATGTCTACAGGTGTGCCTTCGG -3′
**VAMP3 ®**	5′- TTAAGAGACACACCACACGATGATG -3′
**VAMP7 ®**	5’- CCGAGCTCATGGCCATTCTTTTTGCCGTTG -3’
**VAMP7 (F)**	5’- GGAATTCGTTTCTTCACACAGCTTGGACC -3’
**SNAP23 (F)**	5’- TTTCCTGATAAGTTCCTAAATTCCA -3’
**SNAP23 ®**	5’- AAGGCTCTCTCACTCCTCCA -3’
**YKT6 (F)**	5’- GCGATCGCCGGAAACAAAACTCATGCT -3’
**YKT6 ®**	5’- GTTTAAACCCCTGAAGCACAAAGAAAGC -3’
**β-Actin (F)**	5’- CAGGGCGTGATGGTGGGCA -3’
**β-Actin ®**	5’- CAAACATCATCTGGGTCATCTTC -3’

### Western blots

Total proteins were isolated from cells or exosomes using the ReadyPrep™ Protein Extraction Kit (Bio-Rad Laboratories‎, Hercules, CA, USA). Western blots assay was conducted as previously described [61]. The primary antibodies used were as follows: TSG101 (1:1000), CD63 (1:1000), RAB7 (1:500), and β-Actin (1:5000) (Applied Biological Materials Inc., Richmond, BC, Canada). Optical densities of protein bands were determined using Imagej [62].

### Immunofluorescence

Cells (2 × 10^6^) were fixed using 4% paraformaldehyde at room temperature for 25 minutes and then stained with corresponding primary antibodies at 4 °C overnight. The primary antibodies used were as following: ras-related protein Rab-7 (RAB7) (1:100), CD63 (1:200), YKT6 v-SNARE homolog (YKT6) (1:100), and vesicle-associated membrane protein 3 (VAMP3) (1:100) (Applied Biological Materials Inc., Richmond, BC, Canada). The 2-(4-amidinophenyl)-1H-indole-6-carboxamidine (DAPI) was used to stain the nuclei (Thermo Fisher Scientific, Waltham, MA, USA). Immunofluorescence was imaged using the Nikon PCM-2000 confocal microscope (Minato, Tokyo, Japan).

### Pull-down assay

The pull-down assay was performed using the Pierce™ Biotinylated Protein Interaction Pull-Down Kit (Thermo Fisher Scientific, Waltham, MA, USA) according to the manufacturer’s instructions. Biotin-labeled PVT1 or antisense RNA was cocultured with the total protein of HS766T cell and magnetic beads. Western blots were performed to determine RAB7 levels.

### RNA immunoprecipitation (RIP) assay

The RIP assay was performed using Magna RIP™ RNA-Binding Protein Immunoprecipitation Kit (Sigma-Aldrich, Shanghai, China) according to the manufacturer’s instructions. The RAB7 antibody or IgG (negative control) was used to conjugate with magnetic beads loaded with cell lysates. Real-time PCR was performed to determine the level of PVT1 in immunoprecipitated RNAs.

### Palmitoylation assay

The palmitoylation of YKT6 was determined using the palmitoylation assay as previously described [35]. Samples were tested by SDS/PAGE, and the gels were stained with Coomassie Brilliant Blue G-250 Dye (Thermo Fisher Scientific, Waltham, MA, USA).

### Statistical analysis

Statistical analysis was performed using the SPSS 17.0 software (SPSS, Chicago, USA). Data were expressed as mean ± standard deviation (SD). At least three replicates were included in each independent experiment. Student’s t-test and ANOVA were used for statistical analysis. Statistical significance was regarded as *P* < 0.05.
